# Genetic origin and composition of a natural hybrid poplar *Populus × jrtyschensis* from two distantly related species

**DOI:** 10.1186/s12870-016-0776-6

**Published:** 2016-04-18

**Authors:** Dechun Jiang, Jianju Feng, Miao Dong, Guili Wu, Kangshan Mao, Jianquan Liu

**Affiliations:** State Key Laboratory of Grassland Agro-Ecosystem, School of Life Sciences, Lanzhou University, Lanzhou, Gansu People’s Republic of China; College of Plant Sciences, Xinjiang Production & Construction Corps Key Laboratory of Protection and Utilization of Biological Resources in Tarim Basin, Tarimu University, Alar, Xinjiang People’s Republic of China

**Keywords:** Hybrid origin, First-generation hybrids, Hybrid superiority, *Populus × jrtyschensis*

## Abstract

**Background:**

The factors that contribute to and maintain hybrid zones between distinct species are highly variable, depending on hybrid origins, frequencies and fitness. In this study, we aimed to examine genetic origins, compositions and possible maintenance of *Populus × jrtyschensis*, an assumed natural hybrid between two distantly related species. This hybrid poplar occurs mainly on the floodplains along the river valleys between the overlapping distributions of the two putative parents.

**Results:**

We collected 566 individuals from 45 typical populations of *P.* × *jrtyschensis*, *P. nigra* and *P. laurifolia*. We genotyped them based on the sequence variations of one maternally inherited chloroplast DNA (cpDNA) fragment and genetic polymorphisms at 20 SSR loci. We further sequenced eight nuclear genes for 168 individuals from 31 populations. Two groups of cpDNA haplotypes characteristic of *P. nigra* and *P. laurifolia* respectively were both recovered for *P.* × *jrtyschensis*. Genetic structures and coalescent tests of two sets of nuclear population genetic data suggested that *P.* × *jrtyschensis* originated from hybridizations between the two assumed parental species. All examined populations of *P.* × *jrtyschensis* comprise mainly F_1_ hybrids from interspecific hybridizations between *P. nigra* and *P. laurifolia.* In the habitats of *P. × jrtyschensis*, there are lower concentrations of soil nitrogen than in the habitats occupied by the other two species.

**Conclusions:**

Our extensive examination of the genetic composition of *P. × jrtyschensis* suggested that it is typical of F_1_-dominated hybrid zones. This finding plus the low concentration of soil nitrogen in the floodplain soils support the F_1_-dominated bounded hybrid superiority hypothesis of hybrid zone maintenance for this particular hybrid poplar.

**Electronic supplementary material:**

The online version of this article (doi:10.1186/s12870-016-0776-6) contains supplementary material, which is available to authorized users.

## Background

Interspecific hybridization occurs frequently in plants [1–3]; hybrid swarms or hybrid zones provide a window through which to examine species cohesiveness, interspecific gene flow and hybrid fitness [4]. However, it is still hotly debated how such hybrid zones are maintained, mainly because of conflicting views about the relative role of selection versus gene flow in driving or homogenizing divergence [5]. Up to now, three types – tension zones, bounded hybrid superiority zones, and mosaic hybrid zones – have been tentatively suggested, based on theoretical and empirical studies of how selection acts on hybrids and parent species [6, 7]. Within tension zones, hybrids are of low fitness relative to parent species and hybrid zones are restricted to a narrow area between the two parents and are mainly maintained by a balance between dispersal and selection against hybrids [8]. The bounded hybrid superiority (also called the environment-dependent) model assumes that hybrids are fitter than their parents in intermediate habitats, but less fit than parent species in their respective native habitats [9–11]. Gene flow can also be prevented if hybridization proceeds only to the F_1_ stage and no further, which can occur due to apparent habitat-mediated superiority of F_1_s over other hybrid classes [12]. These hybrid zones probably occupy distinct habitats located in an intermediate position, where the ranges of the two parent species overlap. Finally, the mosaic hybrid zone model hypothesizes that patchy environments within the overlapping region of two parent species are highly heterogeneous [7, 13]. Therefore, hybrids comprise a mosaic of diverse genotypes that are highly variable according to their respective distributions. In such a model, both environment-independent and -dependent selections against hybrids co-exist, thus combining the hypotheses of both the tension zone model and the bounded hybrid superiority model. Whichever model applies, it is very important to know the genetic composition of such hybrid zones, with regard to genotype frequencies, before we can identify the factors that may contribute to maintaining these hybrid populations as a result of either intrinsic or extrinsic fitness.

Hybridization and gene flow between species occur extensively in the genus *Populus*, resulting in numerous natural hybrid zones [14–17]. In both Europe [15, 16, 18] and North America [14, 17, 19], the origins of numerous such hybrids have been explored. Some natural poplar hybrid zones contain a mix of F_1_s, post-F_1_s (F_2_s) and further backcross genotypes with diverse levels of fitness [14, 17, 19–21], consistent with a combination of the hybrid tension and superiority hypotheses. Moreover, some natural poplar hybrid zones play a significant role in bridging or preventing gene flow between hybridizing species [14–22]. However, little attention has, so far, been paid to natural hybrids occurring in Asia. In this study, we aimed to examine the genetic origin, composition and possible maintenance of the hybrid between *Populus nigra* and *P. laurifolia* at numerous locations in western China. *Populus nigra*, the black poplar of sect. *Aigeiros*, is mainly found in Europe and has limited ranges in central Asia and northwest Africa [23, 24]. It is, however, a tree of social and economic importance [25]. In western China, it occurs on wet slopes beside rivers at altitudes between 400 m and 1000 m [26]. In contrast, *P. laurifolia* of sect. *Tacamahaca* occurs mainly in northern Asia, with its range extending into central Asia [27]. This species grows on the mountainous slopes of river valleys in western China; it prefers relatively dry habitats at altitudes between 400 m and 1800 m [27]. Despite their distant relationship, as revealed in all phylogenetic studies [28, 29], these two poplars co-occur in Xinjiang, western China. Both of them flower and set seed from April to May [27]. However, these two species differ from each other with respect to numerous characters from leaves to branches and flowers [27]. Both species are dioecious, with pollen dispersed by wind and seeds dispersed by wind and water [30]. They also propagate vegetatively from broken branches and cuttings [31]. Due to their overlapping distributions and flowering periods in western China, a hybrid, *P.* × *jrtyschensis*, was assumed to result from crosses between these two distantly related poplars in Xinjiang [27, 32]. This hybrid and its two putative parent species are diploid with 2n = 38 [27]. It annually sets numerous seeds with unknown fertility [27]. This hybrid poplar has an intermediate morphology between *P. nigra* and *P. laurifolia*, although the overall morphology seems to be more similar to the former than the latter [27, 32]. *P.* × *jrtyschensis* forms pure forests in numerous locations on the floodplains along the Erqis river valley, where neither parent species is present [27, 32]. In addition, this hybrid poplar has been introduced and widely cultivated along agricultural drainage channels, by means of cuttings taken from wild populations, because of its fast growth, straight stems and the other superior characteristics compared to the putative parent species [27, 32].

In addition to the morphological evidence, genetic evidence based on sequence variations from ITS and chloroplast DNA (cpDNA) from samples of several individuals of most species found in Xinjiang has also suggested that *P.* × *jrtyschensis* probably originated from hybridizations between these two distantly related species [33]. We extended the example to include more natural populations of *P.* × *jrtyschensis* and its two putative parental species for the present study. We genotyped a total of 566 individuals from 45 populations of three taxa [see Additional file 1] based on sequence variations of the maternally inherited cpDNA and polymorphisms generated by 20 nuclear simple sequence repeat (SSR) markers. We also sequenced eight nuclear genes for 168 individuals from 31 populations. In this study, we mainly aimed to test the following hypotheses. First, *P.* × *jrtyschensis* originated through hybridization from two distantly related poplar species. This was investigated by examining cpDNA sequence variations and conducting coalescent analyses of genetic polymorphisms from 20 SSR and eight nuclear genes. Second, all examined populations of *P.* × *jrtyschensis* have the same hybrid genetic compositions probably comprised of F_1_s, despite their mosaic distributions due to the relative stability in the morphology of all *P.* × *jrtyschensis* populations. Finally, habitat-selection contributed to the formation of these hybrid swarms and maintained them (bounded hybrid superiority hypothesis) because the floodplains where *P.* × *jrtyschensis* occurs is obviously poorer than the habitats of the two putative parent species. In order to confirm this, we measured and compared the soil nitrogen concentrations in typical habitats of the three taxa.

**Fig. 1:**
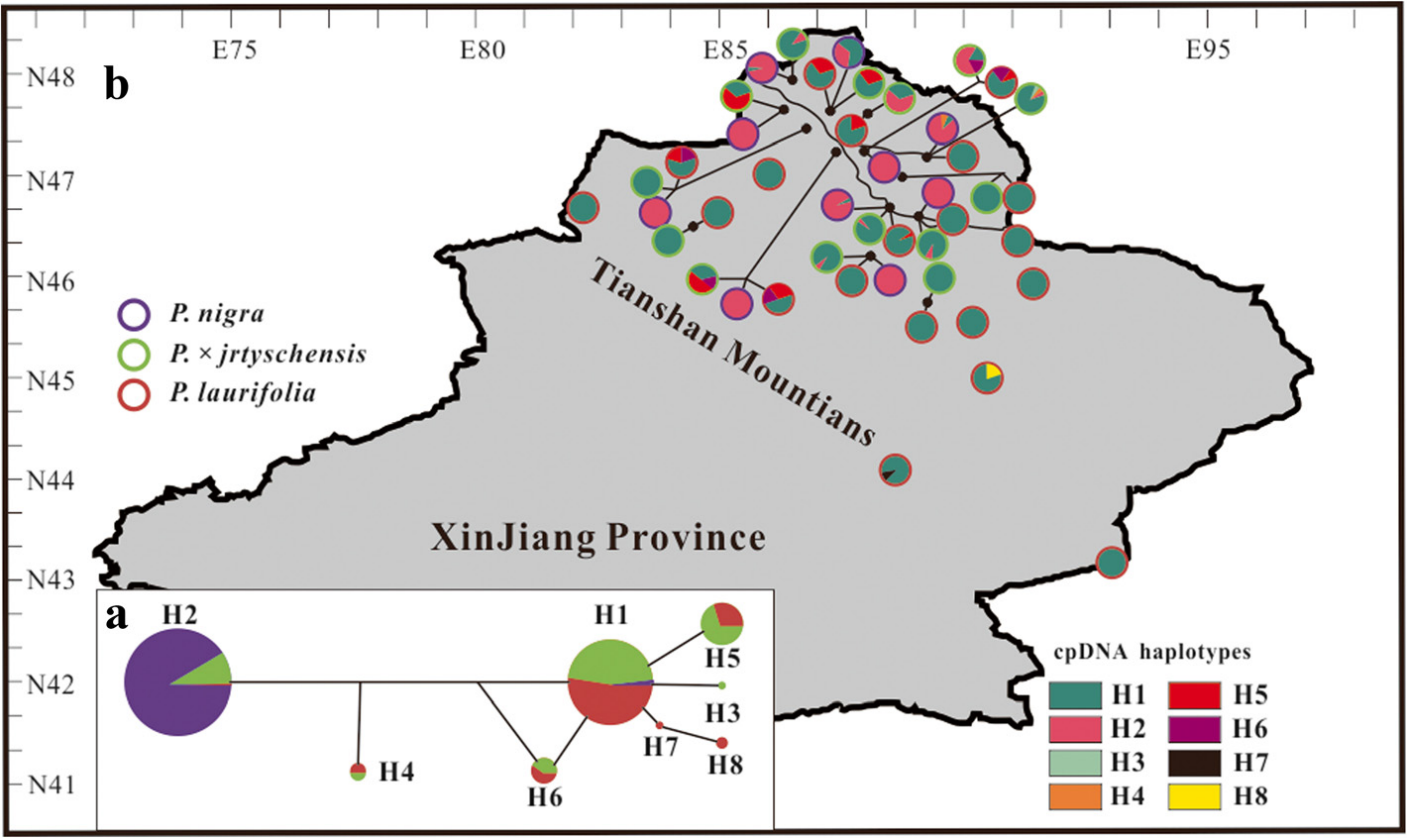
Distribution of haplotypes within the three *Populus species*. **a** Median-joining network among plastid DNA haplotypes present in *P. nigra*, *P. laurifolia* and *P. × jrtyschensis*. Each sector of a circle is proportional to the frequency of each species in each haplotype. Colors of circles in (**b**) indicate the species present at a site. In (b) the sectors of circles indicate the frequency of a haplotype in a population at that site

## Results

### Sequence variation of chloroplast DNA

Thirteen substitutions were detected at the *rbc*L gene across the 566 individuals sampled. These mutations together revealed eight haplotypes (H1-H8, [see Additional file 2]), which clustered into two major groups (Fig. 1): one comprising H2 and the other consisting of H1, H3 and H5-H8. Based on the sequence variations, H4 originated from the recombination of two dominant haplotypes H1 and H2 of the two major groups. Most individuals of *P. nigra* and *P. laurifolia* were found to be fixed into a separate group of haplotypes according to species. For example, H2 was associated with most individuals of *P. nigra* but only one individual of *P. laurifolia*. In contrast, most individuals of *P. laurifolia* were H1, while this haplotype was found for only seven individuals of *P. nigra*. In addition, a few rare haplotypes (H3-H8) were found to be mainly associated with *P. laurifolia*. The individuals of *P. × jrtyschensis* that we examined were found to be represented by five haplotypes of both groups, H1, H2, H4, H5 and H6. Around 94 % of the individuals of *P. × jrtyschensis* were found to have the haplotypes mainly associated with *P. laurifolia* while 6 % were H2, which is mainly found in *P. nigra*. Genetic partitions estimated by AMOVA based on these haplotypes revealed that between-population variation was significant and accounted for 34 % of the total variation in *P. nigra*, but was not significant in *P. laurifolia* where it accounted for only 6 % of the total variation. Between-population differentiation associated with cpDNA sequence variation was significant in *P. × jrtyschensis* and accounted for 28 % of the total variation (Table 1).

**Table 1:** Analyses of molecular variance (AMOVA) for three poplar species based on datasets for two markers

Grouping of regions	Source of variation	d.f.	SS	VC	Percent variation	Fixation index
*rbc*L haplotypes						
All samples	Among groups	2	734.907	1.925	83.32	F_CT_ = 0.8332
	Among populations within species	43	65.524	0.101	4.38	F_ST_ = 0.877
	Within populations	532	151.200	0.284	12.30	F_SC_ = 0.2327
*P. nigra*	Among populations	10	20.304	0.137	34	F_ST_ = 0.34
	Within populations	135	35.799	0.265	66	
*P.* × *jrtyschensis*	Among populations	13	40.935	0.183	28.87	F_ST_ = 0.289
	Within populations	201	90.535	0.45	71.73	
*P. laurifolia*	Among populations	20	4.285	0.009	6.29	F_ST_ = 0.063
	Within populations	196	24.867	0.127	93.71	
SSR markers						
All samples	Among species (average)	2	1535.34	2.013	28.32	F_CT_ = 0.2832
	Among populations within species	42	678.942	0.470	6.62	F_ST_ = 0.3494
	Within populations	1087	5026.09	4.624	65.06	F_SC_ = 0.0923
*P. nigra*	Among populations	10	130.972	0.376	9.22	F_ST_ = 0.0922
	Within populations	271	1003.85	3.704	90.78	
*P.* × *jrtyschensis*	Among populations	13	215.384	0.369	6.15	F_ST_ = 0.0615
	Within populations	418	2349.91	5.622	93.85	
*P. laurifolia*	Among populations	19	332.586	0.639	13.2	F_ST_ = 0.132
	Within populations	398	1672.32	4.202	86.8	
Nuclear genes						
All samples	Among species (average)	2	313.331	1.5804	53.31	F_CT_ = −0.03546
	Among populations within species	29	28.155	−0.04908	−1.66	F_ST_ = 0.51659
	Within populations	272	389.783	1.43303	48.34	F_SC_ = 0.53314
*P. nigra*	Among populations	9	82.674	−0.0513	−0.53	F_ST_ = −0.00534
	Within populations	82	791.608	9.6537	100.53	
*P.* × *jrtyschensis*	Among populations	13	16.811	−0.892	−10.09	F_ST_ = −0.1009
	Within populations	120	1167.883	9.732	110.09	
*P. laurifolia*	Among populations	7	28.565	−1.52901	−8.78	F_ST_ = −0.08785
	Within populations	70	1325.383	18.934	108.78	

*d.f.* degrees of freedom, *SS* sum of squares, *VC* variance components; F_ST_, variance among populations; F_SC_, variance within populations within groups; F_CT_, variance among groups relative to total variance

### Genetic diversity and structure analyses based on eight nuclear genes

**Fig. 2:**
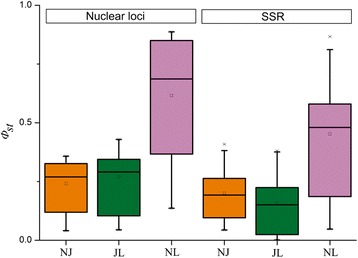
Box plot of genetic differentiation (*Φst*) between each of the three species pairs based on nuclear and SSR data sets. NJ, JL and NL represent the *Φ*_*st*_ value between *P. nigra* and *P. × jrtyschensis*, *P. × jrtyschensis* and *P. laurifolia*, *P. nigra* and *P. laurifolia.* The *Φ*_*st*_ of each locus was estimated individually by AMOVA. Divergence between *P. × jrtyschensis* and parent species is lower than that between parent species as expected for a hybrid species

**Fig. 3:**
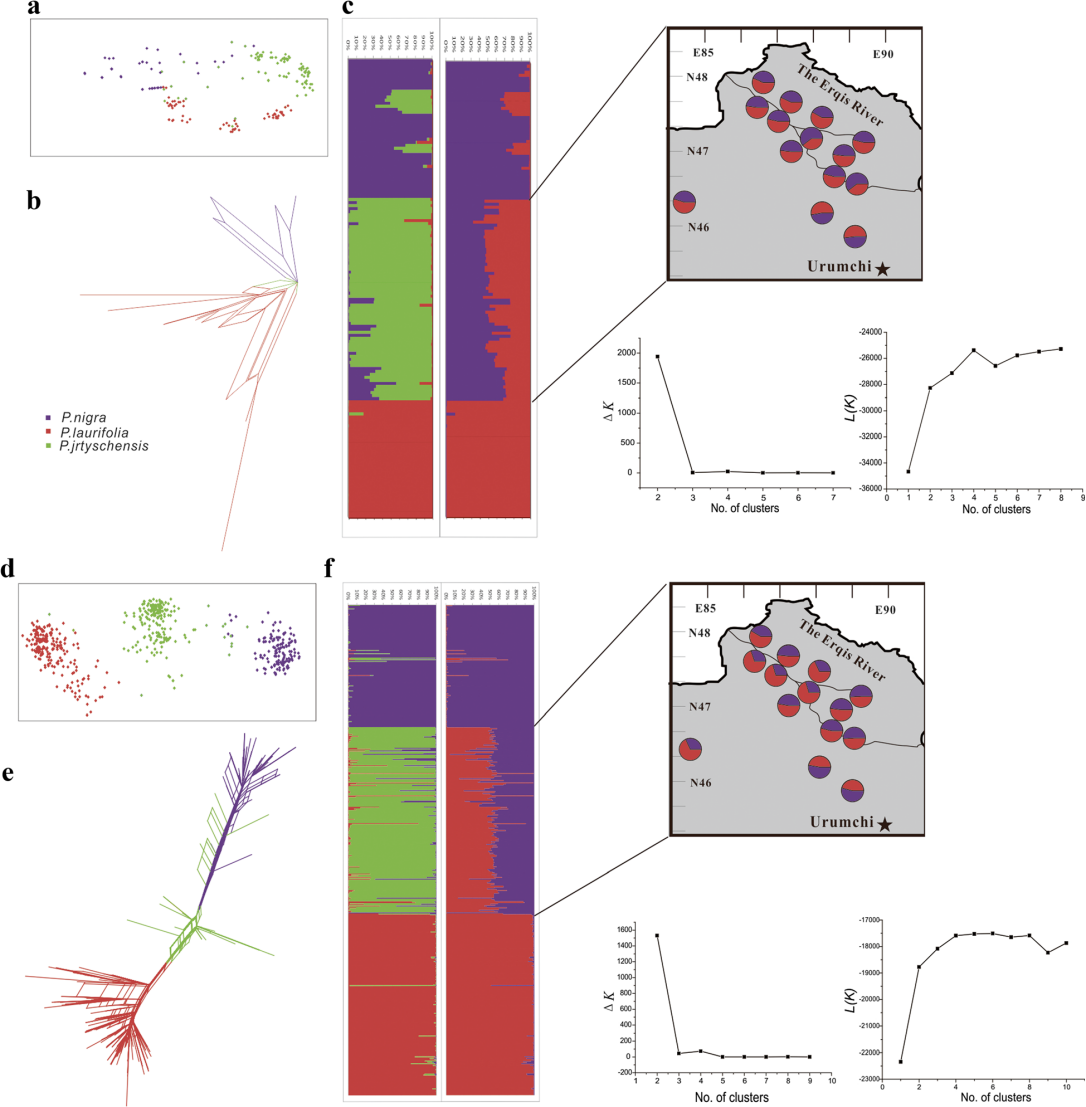
Genetic clustering of all individuals of *P. nigra*, *P. laurifolia* and *P. × jrtyschensis*. **a** Principal Component Analysis (PCA), **b** NeighborNet (NNet), and **c** Population cluster analysis using STRUCTURE (*K* = 2 and 3) based on nuclear gene dataset; **d** Principal Component Analysis (PCA), **e** NeighborNet (NNet), and **f** Population cluster analysis using STRUCTURE (*K* = 2 to 3) based on SSR datasets

Sequence variation and genetic diversity across the eight nuclear loci were both larger in *P. × jrtyschensis* than in the other two species [see Additional file 3]. Private single-nucleotide polymorphisms (SNPs) for each parent species were recovered at each locus, with shared SNPs being more common between *P. × jrtyschensis* and *P. nigra* than between *P. × jrtyschensis* and *P. laurifolia* (Table 2). Both PCAs of samples and the NNet tree constructed for all samples suggested a hybrid origin of *P. × jrtyschensis* (Fig. 3a, b). Structure also revealed that when K was set to 2 in Structure with USEPOPINFO = 1, *P. nigra* and *P. laurifolia* individuals clustered into two separate groups, while individuals of *P. × jrtyschensis* were admixed, containing a mixture of the genomes of the two groups representing the putative parent species (Fig. 3c). Both the Pritchard et al. [34] and Evanno et al. [35] tests indicated that the most likely number of clusters for the entire data set was *K* = 2. Genetic divergence between the three taxa further indicated that *P. × jrtyschensis* was a hybrid, in that divergence between *P. × jrtyschensis* and either *P. nigra* or *P. laurifolia* was similar, while pairwise *Φ*_*st*_ values for comparisons between *P. × jrtyschensis* and either *P. nigra* or *P. laurifolia* were lower than between *P. nigra* and *P. laurifolia* (Fig. 2) [see Additional file 4]. In each taxa, the positive values for both Tajima’s D and Fu & Li’s D and F were estimated for half of the nuclear loci and negative for the others [see Additional file 3].

**Table 2:** Distribution of segregating sites at nuclear loci in pairwise comparisons of taxa: *P. nigra*, *P. × jrtyschensis* and *P. laurifolia*

Gene	*P. nigra* vs. *P. × jrtyschensis*				*P. × jrtyschensis* vs *P. laurifolia*				*P. nigra* vs. *P. laurifolia*			
	S_n_	S_j_	S_s_	S_f_	S_l_	S_j_	S_s_	S_f_	S_n_	S_l_	S_s_	S_f_
Dehy	11	14	10	0	3	14	3	0	11	3	1	0
Phyto A	2	3	2	0	0	3	0	0	2	0	0	0
Phyto B	3	4	2	0	1	4	1	0	3	1	0	0
PAL	20	27	20	0	16	27	16	0	20	16	16	2
AREB1	35	39	33	0	34	39	33	0	35	34	31	0
ERD7	6	8	6	0	5	8	5	0	6	5	4	0
EIN3	30	32	30	0	28	32	28	0	30	28	26	0
LTCOR11	5	7	5	0	2	7	2	0	5	2	1	0

S_n_, S_j_ and S_l_ are the number of polymorphic sites unique to *P. nigra*, *P. × jrtyschensis* and *P. laurifolia*, respectively, in each comparison; S_s_ is the number of sites with shared alleles between the two taxa; and S_f_ is the number of sites with fixed alleles in either taxa

### Genetic diversity and structure analyses based on SSR loci

The alleles per locus and the estimated genetic indexes for each of the three taxa were listed in [Additional file 5]. Allelic richness at each locus was higher in *P. × jrtyschensis* than *P. nigra* or *P. laurifolia* [see Additional file 6]. Both PCAs for samples from the three taxa and the NNet tree constructed for all samples based on genetic distance suggested that *P. × jrtyschensis* was located between *P. nigra* and *P. laurifolia*, with a closer affinity with the former than the latter. The *P. × jrtyschensis* cluster was clear (Fig. 3d, e), but both the Pritchard et al. [34] and Evanno et al. [35] tests indicated that the most likely number of clusters for the entire data set was *K* = 2. When K was artificially set to 2, all individuals of *P. × jrtyschensis* were admixed, with a mixture of the genomes of the two groups representing the two parent species (Fig. 3f).

### Test of the hybrid origin and hybrid composition of *P. × jrtyschensis* based on population genetic data from 20 SSRs and eight nuclear genes

**Fig. 4:**
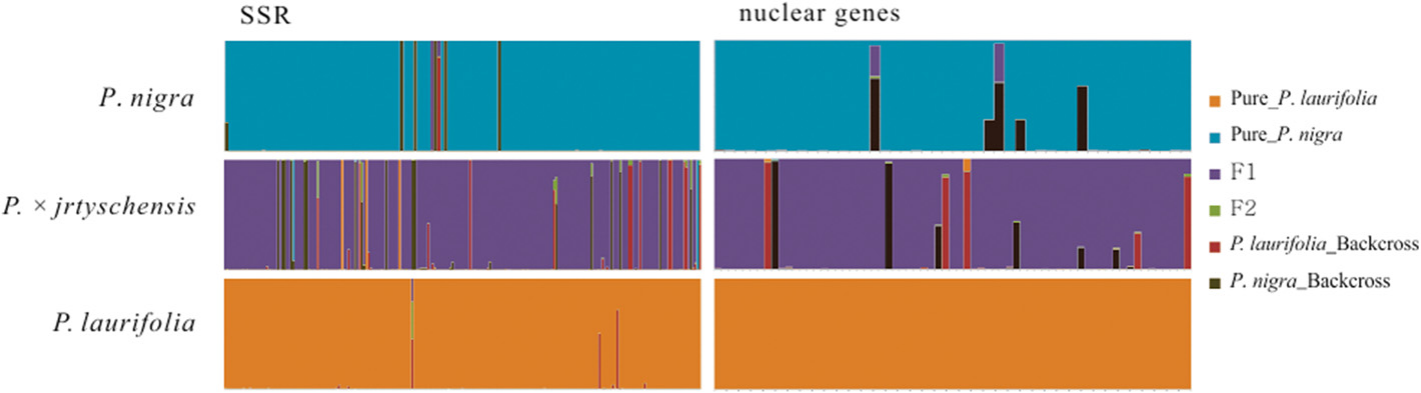
Estimated posterior probabilities for each individual being pure parents, F_1_, F_2_ and backcross genotypes. The height of the column for each individual represents the probability of a single frequency class. An individual was considered assigned if the probability of a single frequency class exceeded 90 %

**Fig. 5:**
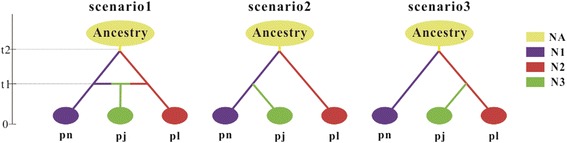
Scenarios that were tested for the origin of *P. × jrtyschensis* (pj), *P. nigra* (pn) and *P. laurifolia* (pl). N1, N2 and N3 represent current effective population sizes of *P. nigra*, *P. laurifolia* and *P. × jrtyschensis*, respectively. For Scenarios 1–3, t1 is the time of origin of *P. × jrtyschensis*. t2 represents divergence time between *P. nigra* and *P. laurifolia* in “generations ago” and NA is the effective population size of the common ancestor of the three species

We tested three alternative divergence hypotheses for the three taxa based on SSR and nuclear gene data sets separately (Fig. 5). Our ABC modeling results revealed that the hybrid origin model (Scenario 1, Fig. 5) provided a better fit for the observed data than Scenarios 2 and 3. The posterior probabilities of Scenarios 1, 2 and 3 were, respectively, 0.978, 0.004 and 0.0216 for SSRs and 0.382, 0.28 and 0.338 for the nuclear sequence dataset [see Additional file 7]. We tested hybrid composition criteria based on NewHybrids estimates suggested by Anderson and Thompson [36] using SSR and nuclear gene data sets. For SSRs, 95 % of the sampled individuals under *P. nigra* and 99 % of the sampled individuals under *P. laurifolia* were pure. In total, 84 % of the sampled individuals of *P. × jrtyschensis* were considered to be F_1_ hybrids between pure *P. nigra* and *P. laurifolia*. In addition, 6 % of individuals are backcrosses with one of the parents, while it is difficult to ascribe the remaining individuals (Fig. 4). Similarly, based on sequence variations of nuclear genes, 90 and 100 % of the sampled individuals under *P. nigra* or *P. laurifolia* were found to be pure. In addition, 87 % of the sampled individuals of *P. × jrtyschensis* were considered to be F_1_ hybrids while 9 % of them seems to be backcrosses with one of the parents and the remaining individuals were difficult to ascribe. Only two individuals from one population were found to have the same marked polymorphisms at all 20 SSR loci, suggesting that they derived from the same clone. No single clone was found in any two different populations.

**Fig. 6:**
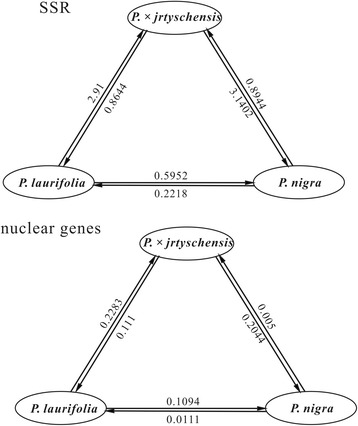
Gene flow for all three species pairs. Gene flow for all three species pairs is represented by arrows. Figures beside the arrows indicate the population migration rate (N_e_m)

Based on SSR data sets, gene flow (N_e_m) was estimated to be greater from *P. laurifolia* and *P. nigra* (0.5952) than in the opposite direction (0.2218). Gene flow occurred more frequently between *P. × jrtyschensis* and the two parent species. More gene flow occurred from *P. laurifolia* to *P. × jrtyschensis* (2.91) than in the reverse direction (0.8644) while less was detected from *P. nigra* (0.8944) to *P. × jrtyschensis* than in the reverse direction (3.1402). The same trend was observed based on nuclear genes: gene flow was estimated to be 0.1094, 0.005 and 0.111 separately from *P. laurifolia* to *P. nigra*, from *P. nigra* to *P. × jrtyschensis* and from *P. × jrtyschensis* to *P. laurifolia*, respectively, and in the opposite direction it was estimated to be 0.0111, 0.2044 and 0.2283. In all directions, rates of gene flow estimated for the SSR data set were greater than those based on nuclear gene sequence data (Fig. 6).

### Soil nitrogen analyses of typical habitats for three taxa

**Fig. 7:**
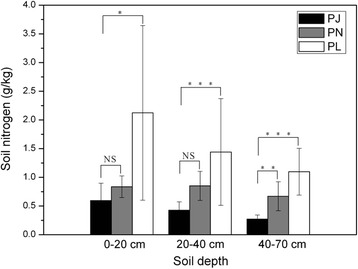
Comparisons of soil nitrogen concentration at each depth for the three taxa’s sites. Error bars represent SE. PJ, *P. × jrtyschensis*; PN, *P. nigra*; and PL, *P. laurifolia*. Significant differences in soil nitrogen concentration at each depth for the sites representing the habitat of each of the three taxa as revealed by ANOVA: NS not significant, *P* > 0.1; *P < 0.05; **P < 0.01; ***P < 0.001

Total soil nitrogen concentration of typical habitats of *P. × jrtyschensis* differed from those of the two parent species. The typical habitats of *P. × jrtyschensis* had lower nitrogen concentrations at depths of 0–20 cm, 20–40 cm and 40–70 cm than the habitats of the two parent species (Fig. 7). In addition, we found that soil nitrogen concentrations were significantly different between *P. × jrtyschensis* habitats and the habitats of the two parent species, with higher probabilities for the greater depths [see Additional files 8, 9 and 10].

## Discussion

In this study, we used 20 SSR markers, eight nuclear gene markers and cpDNA sequence variations to genotype 566 individuals from 45 populations of *P.* × *jrtyschensis*, *P. nigra* and *P. laurifolia*. In addition to the intermediate morphology of the hybrid compared to the two putative parents [27, 32], our genetic results provided further support for the hypothesis that *P. × jrtyschensis* originated from hybridizations between the distantly related species *P. nigra* and *P. laurifolia*. Our reasons for this conclusion are as follows. First, the detected alleles for each individual of *P. × jrtyschensis* were admixed with the clusters specific to the putative parent species. That the species-specific alleles co-occurred in one taxa undoubtedly suggested its hybrid origin [34]. This scenario has been confirmed in some case study of hybrid taxa [12]. Second, ABC analyses supported the hybrid origin hypothesis for *P. × jrtyschensis* while the alternative hypotheses suggesting divergences from one of the two parent species were rejected (Fig. 5). Finally, two distinct cpDNA lineages were recovered for *P. nigra* and *P. laurifolia* respectively while both of them co-occurred in *P. × jrtyschensis*. Two divergent maternal lineages from putative parents have also been reported for other hybrid taxa [2, 3]. These lines of evidence together supported the hypothesis that *P. × jrtyschensis* originated from hybridizations between *P. nigra* and *P. laurifolia.*

Further, we found that most of the populations of *P. × jrtyschensis* that we examined comprised F_1_ hybrids with a few backcrosses with each of the two parent species, although clonal reproduction did occur in some of them. These findings did not support the other two original hypotheses regarding the intermediate but stable morphology of *P. × jrtyschensis*, namely that they either derived from a few clonal lineages or had developed into a stable homoploid hybrid species. However, in a typical hybrid zone, F_1_s usually comprise a very small number of the individuals present [37, 38]. Relatively few hybrid zones have been reported to be dominated by F_1_s; those that were known include *Encelia* × *laciniata* [39], the hybrid zone between Black Oaks [40], *Rhododendron* × *sochadzeae* [12] and *Rhododendron agastum* [41]. A predominance of F_1_s has rarely been found in hybrid swarms between other *Populus* species and most hybrid swarms contain F_1_s, F_2_s as well as backcrosses [14, 17, 19–21]. In a previous study [14], only F_1_s were detected between *P. deltoides* and *P. nigra*, possibly due to their distant relationship and strong reproductive isolation. According to our field observations, *P. × jrtyschensis* produced numerous seeds. However, it remains unknown whether these seeds germinate. We also failed to find young seedlings from the habitat of *P. × jrtyschensis*, which seems to support the conclusion that the populations of *P. × jrtyschensis* mainly comprise F_1_s. Because we did detect backcross hybrids (although fewer individuals) with both *P. nigra* and *P. laurifolia*, pollen-stigma incompatibility is unlikely to account for the general absence of the post-F_1_s in most of the populations of *P. × jrtyschensis* that we examined. However, introgressions between *P. nigra* and *P. laurifolia* are relatively small according to our estimations based on the nuclear dataset (Fig. 6) despite the fact that these F_1_s might have resulted from the repeated hybridizations between two parental species.

The presence of these mosaic hybrid populations consisting mainly of F_1_s suggests two alternative origins: a recent contact between two parental species only one generation ago without enough time for post-F_1_ derivatives to have been produced or that these F_1_s may exclude other genotypes from the hybrid habitats [12, 37]. Numerous individuals of each examined population are at least 50 years old according to rough estimates based on their large stems compared with other poplars encountered during our field surveys. Although accurate data on flowering age of *P. × jrtyschensis* are not available, this should be similar to other poplars, i.e. between 10 and 30 years [27]. Therefore, most genets of each population should have existed long enough for post-F_1_ progeny to have been produced. Thus, it appears that the *P. × jrtyschensis* populations comprise stable and long-lived hybrid zones dominated by F_1_s, and other genotypes were excluded because of the habitat selection. The distributional preferences of *P. × jrtyschensis* and the two parent species also support this habitat-selection suggestion. At a local scale, *P. × jrtyschensis* is parapatric, rather than strictly sympatric to the two parent species. One of the parent species, *P. nigra*, was found on wet slopes adjacent to rivers, whilst the other, *P. laurifolia*, was found on dry mountainous slopes; in contrast, *P. × jrtyschensis* occurs exclusively on the floodplains. Three examined sites with *P. × jrtyschensis* were found to be nutrient-poor with low concentrations of the total soil nitrogen, especially in the deeper layers (Fig. 7). Such differentiations of the habitat preferences have also been noted between some hybrid taxa and their respective parental species for other plant genera [12, 39, 41]. The habitat-mediated selection may have prevented other genotypes (parents, BCs and F_2_s) from germination and surviving in the floodplains occupied by *P. × jrtyschensis*. In addition, new and recent hybridizations between two parental species may have continuously produced more F_1_s to repopulate the *P. × jrtyschensis* hybrid zones. It is highly likely that habitat-mediated selection as well as repeated productions of the F_1_s between two parental species have together maintained the unique F_1_ hybrid zones detected here.

Although direct comparisons of fitness between F_1_s and F_2_s or further backcrosses with either parent are rarely undertaken [6], a higher fitness for F_1_s is theoretically likely. Complete gene sets from both parents are present in F_1_s, and heterosis and hybrid vigor undoubtedly persist without hybrid breakdown [42, 43]. All beneficial traits conferred through the co-adapted gene complexes from two parents can be passed intact to the F_1_ generation, but not to post-F_1_s because such gene complexes are likely to be broken down. Therefore, if some of these co-adapted gene complexes confer a benefit to F_1_s through heterosis when occupying new niches, then these effects will be reduced in post-F_1_s due to the lower proportion of heterozygous loci, reflecting post-mating reproductive isolation between highly divergent species. However, increased fitness in the post-F_1_s could derive from transgressive segregations, which give rise to beneficial traits that do not exist in the parent species, in homoploid hybrid neospecies or in plants developing into independent lineages [42, 43]. Theoretically, some post-F_1_s are likely to develop superior traits over F_1_s to occupy novel or arid habitats in places that do not favor F_1_s, but which neither of the parents are adapted to. This may be true for *P. × jrtyschensis* although the predominance of F_1_s in the patchy habitat prevents further segregations. In addition, the backcross frequencies observed here are extremely low, although we could not exclude the possibility that this was the result of widespread and strong genomic incompatibility between these highly divergent species. It is also likely that further backcross hybridizations were excluded by unfavorable epistatic combinations that led to unfit progeny. All these hypotheses and those suggesting higher fitness of the F_1_s than F_2_s, BCs and parents need further artificially controlled tests especially in the soils with the limited nitrogen concentration, as have recently been undertaken for spruce hybrids [44], before definitive conclusions can be drawn.

## Conclusions

Our results suggest that *P. × jrtyschensis* is typical of F_1_-dominated hybrid zones between the distantly related species *P. nigra* and *P. laurifolia*. Habitat-mediated selection due to F_1_ superiority as well as continuous production of the more F_1_s due to the repeated hybridizations between two parental specie are likely to have maintained these hybrid populations. Therefore, the formation of *P. × jrtyschensis* hybrid zones is largely consistent with the environment-dependent bounded hybrid superiority hypothesis. In addition, because of the absence of a basic difference in the genetic composition between the populations of *P. × jrtyschensis* examined, individuals for cultivation of this hybrid poplar can be obtained from vegetative cuttings from any natural population.

## Methods

### Ethics statement

All leave samples employed in this study were collected from tree species that are not endangered, and these trees grow in public area where no permission for collection of leaves is needed in China. All soil samples employed in this study were collected from public area where no permission is needed in China.

### Sampling and sequencing

Leaves of 566 samples were collected from 45 populations of *Populus × jrtyschensis*, *P. nigra*, and *P. laurifolia* in Xinjiang, western China [see Additional files 1 and 11]. These populations cover the distributional ranges of *P. nigra* and *P. laurifolia* in Xinjiang, within which *Populus × jrtyschensis* occurs. Trees from each population (or location) were randomly sampled and an effort was made to avoid sampling closely related individuals or clones. Fresh leaves were dried and stored in silica gel, and the latitude, longitude and altitude of each collection site were recorded using an eTrex GIS unit (Garmin, Taiwan). We extracted the total DNA using the modified hexadecetyltrimethyl ammonium bromide (CTAB) procedure [45, 46]. Following DNA extraction, a total of nine DNA fragments were amplified and sequenced. These sequences included one chloroplast gene *rbc*L (for 566 individuals) and eight nuclear genes (*Dehy*, *PhytoA*, *PhytoB*, *PAL*, *AREB1*, *ERD7*, *EIN3* and *LTCOR11*) (for 168 individuals from 31 populations) [see Additional file 12]. The nuclear genes were selected and primers were designed from the genome sequences of two poplars (*Populus euphratica* Oliv. and *P. trichocarpa* Torr.) [47]. Sequences were edited and aligned manually using MEGA5 [48]. All newly obtained sequences for each taxon have been deposited in GenBank. All polymorphic and heterozygous sites were visually confirmed and separated. We further examined genetic polymorphisms of all 566 samples using 20 pairs of nuclear simple sequence repeat (SSR) primers reported before [see Additional file 13] [47, 49],

### Population genetic analyses

We determined basic population genetic parameters for the eight nuclear genes using DnaSP, version 5.0 [50], after excluding insertions/deletions (indels). We estimated the number of segregating sites (S), Watterson’s parameter (θ_w_) [51], nucleotide diversity (π, [52]) and the minimum number of recombinant events (R_m_, [53]). Haplotypes were investigated by estimating haplotype number (K) and diversity (H_d_) for each gene based on the number of segregating sites [54, 55]. We tested the neutral evolution of loci using diverse statistics, including Tajima’s D statistic [56], Fu and Li’s D*and F* [57]. To quantify the extent of genetic divergence between species, we calculated the fixation index *Φ*_*st*_ [58], based on population genetic data for the eight nuclear loci and the 20 SSRs using ARLEQUIN version 3.0 [59], with significance determined by permutation tests involving 10,000 resamples. ARLEQUIN v.3.0 [59] was also used to quantify hierarchical genetic divergence between and within species using an AMOVA analysis based on nuclear data for the eight nuclear loci and the 20 SSRs; significance was assessed using the permutation test in the program with 1000 permutations. The NETWORK program [60] was used to construct a network of relationships between haplotypes identified for each nuclear locus and also to construct a network of cpDNA haplotypes based on sequence variation across *rbc*L fragments. The default settings were used for all other parameters.

The Bayesian model-based clustering method in STRUCTURE version 2.3.2 [35, 61] was used to examine genetic clustering of the nuclear data. In the analysis of nuclear sequence variation, only individuals (*N* = 155) with sequences for all eight loci and that were satisfactorily phased were included, while the analysis of SSR genotypes included all individuals (*N* = 566). To assign individuals to genetic groups (K), 10 replicate runs were conducted for each value of K, ranging from 1 to 10. The admixture model with correlated allele frequencies was used for each run with no prior placed on population origin. Each run included a burn-in of 500,000 followed by 2,000,000 Monte Carlo Markov chain (MCMC) iterations. The most likely number of clusters was estimated using the original method from Pritchard et al. [34] and also theΔK statistic of Evanno et al. [35]. Graphics were produced using Origin version 8.

To detect genetic groupings further, principal component analysis (PCA) was also conducted separately on the nuclear gene and SSR data sets (using GenAlEx 6.5 [62]). We also used the Neighbor-Net algorithm (NNet) [63] within SPLITSTREE version 4.13.1 [64] to construct the phylogenetic relationships between individuals based on 11 of the nuclear genes and the SSR data set. NeighborNet networks were used to provide more detailed visualization of any potential conflicts among the analyzed genotypes. These conflicts can be the result of evolutionary events such as hybridization, polyploidization and recombination [65, 66]. The genetic distances based on the SSR data set were measured by GenAlEx 6.5 [62].

### Test of the hybrid origin and hybrid composition of *P. × jrtyschensis*

Three alternative divergence and speciation histories hypothesized for the three taxa were summarized in Fig. 5. We used population genetic data obtained from eight nuclear gene sequences and 20 SSR markers to test which of these three models provided the best fit for the data using Approximate Bayesian Computation (ABC) analysis in DIYABC, version 2.0.4 [67, 68]. We set the order of evolutionary relationships between *P. × jrtyschensis*, *P. nigra*, and *P. laurifolia* using uniform population-size parameters and timing parameters for dating divergence and hybridization. In the hybridization model, *P. × jrtyschensis* originated from a hybrid population between the other two species. In the other two scenarios, *P. × jrtyschensis* diverged from a common ancestor with one of the other two species. To select the model that best explained the genetic polymorphism observed in the three varieties, 1,000,000 multilocus genetic data sets were simulated for each scenario. We used the 1 % of the simulated data sets closest to the observed data to estimate the relative posterior probability [with 95 % confidence intervals (CIs)] for each scenario via logistic regression and posterior parameter distributions according to the most likely scenario [67, 68]. Mutation rates were assumed to be between 10^−4^ and 10^−3^ substitutions/site/year [69].

In addition, we checked whether each population of *P. × jrtyschensis* comprised an F_1_ generation or further backcrosses with each parent species, using NewHybrids Version 1.0 [36] to estimate posterior probabilities for each individual being pure parental, F_1_, F_2_ or backcrossed genotypes based on the SSR and nuclear gene data sets. An individual was considered assigned if the probability of a single frequency class exceeded 90 %. We assumed that the sampled individuals originated from the same clone if they shared the same genetic polymorphisms at the 20 loci examined. We used Genclone 2.0 to detect clone individuals across all 45 populations.

Finally, we used the coalescent-based program IMa2 [70, 71] to estimate gene flow between the three taxa using the SSR and nuclear genes data sets. The mutation rate was assumed to be 10^−4^ substitutions/site/year [69] and was input as a point estimate. Average generation time was set to 15 years based on previous estimates for poplar trees [72].

### Soil nitrogen analysis

Soil samples were randomly collected by taking 5-cm-diameter soil cores from 0 to 20, 20 to 40 and 40 to 70 cm depths from nine typical sites for the three taxa (three different sites for each taxon, [see Additional file 14]). All samples were dried at 105 °C to constant weight and passed through a 1 mm sieve prior to nitrogen analysis. Total nitrogen of each soil sample was determined using a Nitrogen Analyzer System (KJELTEC 2300 AUTO SYSTEM II). All statistical analyses were carried out in the SPSS statistical software package (SPSS Inc. Chicago, IL, USA). Graphics were produced using Origin version 8.

## Consent to Publish

Not applicable.

## Availability of data and materials

The datasets supporting the conclusions of this article are included within the article and its additional files. The nuclear genes sequences datasets are available in the Genebank repository Accessions no: KT626975-KT629820 [see Additional file 15] [http://www.ncbi.nlm.nih.gov/genbank/].

## Additional files

Additional file 1: The geographical distribution of populations. (PDF 48 kb) Additional file 2: Variable sites of the aligned sequences of chloroplast DNA fragments in eight haplotypes of *P. nigra*, *P. laurifolia* and *P. × jrtyschensis*. (PDF 29 kb) Additional file 3: Nucleotide variation, haplotype diversity and neutrality tests at eight nuclear loci in (a) *P. nigra*, (b) *P. × jrtyschensis*, (c) *P. laurifolia.* (XLS 36 kb) Additional file 4:
*Φ*_*st*_ values for each taxa pair at eight nuclear loci and for the SSR data set. (PDF 147 kb) Additional file 5: Estimates of genetic diversity (per locus) for each species and their hybrid based on 20 microsatellite loci. (XLS 36 kb) Additional file 6: Characteristics of 20 microsatellite loci scored in the 566 individuals. (PDF 17 kb) Additional file 7: Description of all scenarios used in the Approximate Bayesian Computation analysis in DIYABC v2.0.4 to test the hybrid origin. (PDF 164 kb) Additional file 8: Summary results of one-way ANOVAs to determine the significant differences in soil nitrogen concentration at a depth of 0–20 cm for three taxa. (PDF 99 kb) Additional file 9: Summary results of one-way ANOVAs to determine the significant differences in soil nitrogen concentration at a depth of 20–40 cm for three taxa. (PDF 98 kb) Additional file 10: Summary results of one-way ANOVAs to determine the significant differences in soil nitrogen concentration at a depth of 40–70 cm for three taxa. (PDF 173 kb) Additional file 11: Sample information for each population. (XLS 30 kb) Additional file 12: The genes and primers used in this study. (PDF 162 kb) Additional file 13: SSR Primers used in this study. (PDF 99 kb) Additional file 14: The three different sites for each taxon used for the soil nitrogen analysis. (PDF 152 kb) Additional file 15: GenBank Accession numbers for all newly obtained sequences for the three taxa included in this study. (PDF 17 kb)

## Abbreviations

cpDNA: chloroplast DNA
BCs: backcrosses
SMM: stepwise mutation model
IAM: infinite allele model
NNet: NeighborNet
MCMC: Monte Carlo Markov Chain
PCA: principal component analysis
ABC: Approximate Bayesian Computation
