# Supplementary figures and images for: Genetic origin and composition of a natural hybrid poplar Populus × jrtyschensis from two distantly related species
Source: BMC Plant Biol. 2016 Apr 18;16:89. doi: 10.1186/s12870-016-0776-6 (PMC4836070; doi:10.1186/s12870-016-0776-6)

Additional file 15 The geographical distribution of populations.

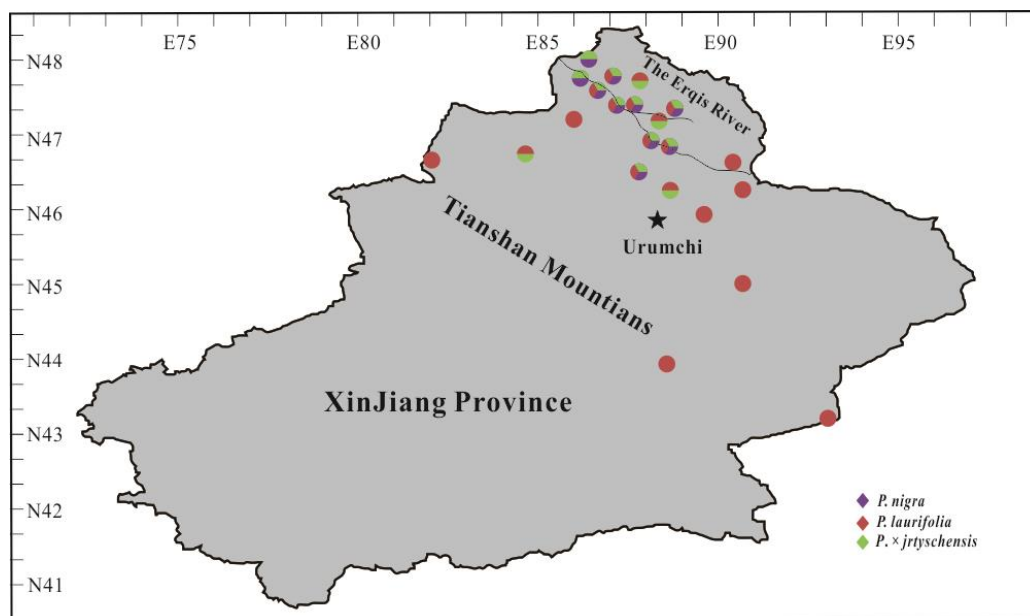

Supplement: Additional file 1: — The geographical distribution of populations. (PDF 48 kb) [file 12870_2016_776_MOESM1_ESM.pdf]
