# Supplementary material for: Genetic origin and composition of a natural hybrid poplar Populus × jrtyschensis from two distantly related species
Source: BMC Plant Biol. 2016 Apr 18;16:89. doi: 10.1186/s12870-016-0776-6 (PMC4836070; doi:10.1186/s12870-016-0776-6)
Supplement: Additional file 2: — Variable sites of the aligned sequences of chloroplast DNA fragments in eight haplotypes of P. nigra, P. laurifolia and P. × jrtyschensis. (PDF 29 kb) [file 12870_2016_776_MOESM2_ESM.pdf]

Additional file 1 Variable sites of the aligned sequences of chloroplast DNA fragment in eight haplotypes of *P. nigra*, *P. laurifolia* and *P.* × *jrtyschensis*.

Chlorotype	*rbc*L												
	1	7	39	50	66	213	243	272	415	670	735	745	781
H1	A	G	T	C	C	T	C	T	T	G	C	G	C
H2	A	A	G	A	A	T	C	T	A	A	G	A	C
H3	A	G	T	C	C	G	C	T	T	G	C	G	C
H4	A	A	T	C	A	T	C	A	A	A	G	A	C
H5	A	G	T	C	C	T	T	T	T	G	C	G	C
H6	A	G	T	C	C	T	C	T	T	G	A	G	C
H7	G	G	T	C	C	T	C	T	T	G	C	G	C
H8	G	G	T	C	C	T	C	T	T	G	C	G	T
