# Supplementary material for: Genetic origin and composition of a natural hybrid poplar Populus × jrtyschensis from two distantly related species
Source: BMC Plant Biol. 2016 Apr 18;16:89. doi: 10.1186/s12870-016-0776-6 (PMC4836070; doi:10.1186/s12870-016-0776-6)
Supplement: Additional file 4: — Φ st values for each taxa pair at eight nuclear loci and for the SSR data set. (PDF 147 kb) [file 12870_2016_776_MOESM4_ESM.pdf]

Table S14 Φ_{st} values for each taxa pair at 8 nuclear loci and for SSR data set.

Φ_{st}	*P. nigra* vs. *P. × jrtyschensis*	*P. × jrtyschensis* vs *P. laurifolia*	*P. nigra* vs. *P. laurifolia*
Dehy	0.2694***	0.29086***	0.68641***
Phyto A	0.29852***	0.26236***	0.75115***
Phyto B	0.35796***	0.42896***	0.86193***
PAL	0.1707***	0.3244***	0.38799***
AREB1	0.11899**	0.10382***	0.36672***
ERD7	0.32606***	0.35706***	0.84995***
EIN3	0.04084***	0.04452***	0.13621***
LTCOR11	0.34529***	0.34446***	0.88673***
PeuSSR_37942	0.19252***	0.1734***	0.42833***
PeuSSR_69373	0.18542***	0.15125***	0.48010***
PeuSSR_82554	0.04582***	0.00177	0.05562***
PeuSSR_98348	0.25472***	0.16250***	0.55249***
PeuSSR_135688	0.20215***	0.14159***	0.48900***
PeuSSR_135862	0.27646***	0.37595***	0.81113***
PeuSSR_149476	0.26314***	0.22424***	0.67514***
PeuSSR_185039	0.26030***	0.00112	0.23852***
PeuSSR_1063	0.21198***	0.12400***	0.48802***
PeuSSR_1065	0.33253***	0.30634***	0.76772***
PeuSSR_114	0.09591***	0.28614***	0.57969***
PeuSSR_1158	0.17326***	0.16689***	0.52311***
PeuSSR_124	0.38168***	0.38093***	0.86655***
PeuSSR_1255	0.15322***	0.21829***	0.46648***
PeuSSR_1260	0.08675***	0.03101*	0.10927***
PeuSSR_11	0.04400***	0.00607*	0.04742***
PeuSSR_186	0.40873***	0.00227	0.34875***
PeuSSR_190	0.28671***	0.31691***	0.78605***
PeuSSR_264	0.10999***	0.02404**	0.18596***
PeuSSR_279	0.04342**	0.07173***	0.15041***

Significance at * $P < 0.05$, **$P < 0.01$ and ***$P < 0.001$.
