# Supplementary material for: Genetic origin and composition of a natural hybrid poplar Populus × jrtyschensis from two distantly related species
Source: BMC Plant Biol. 2016 Apr 18;16:89. doi: 10.1186/s12870-016-0776-6 (PMC4836070; doi:10.1186/s12870-016-0776-6)
Supplement: Additional file 6: — Characteristics of 20 microsatellite loci scored in the 566 individuals. (PDF 17 kb) [file 12870_2016_776_MOESM6_ESM.pdf]

Additional file 3 Characteristics of 20 microsatellite loci scored in 566 individuals

Locus Name	Number of alleles	Allelic richness
PeuSSR_37942	11	2.907
PeuSSR_69373	18	2.959
PeuSSR_82554	43	3.667
PeuSSR_98348	20	2.742
PeuSSR_135688	29	2.904
PeuSSR_135862	7	2.09
PeuSSR_149476	13	2.386
PeuSSR_185039	34	2.767
PeuSSR_1063	17	2.937
PeuSSR_1065	5	2.008
PeuSSR_114	5	1.712
PeuSSR_1158	11	2.588
PeuSSR_124	5	2.016
PeuSSR_1255	14	2.633
PeuSSR_1260	10	2.696
PeuSSR_11	24	1.57
PeuSSR_186	25	2.165
PeuSSR_190	18	2.43
PeuSSR_264	24	1.368
PeuSSR_279	40	3.49
