# Supplementary material for: Genetic origin and composition of a natural hybrid poplar Populus × jrtyschensis from two distantly related species
Source: BMC Plant Biol. 2016 Apr 18;16:89. doi: 10.1186/s12870-016-0776-6 (PMC4836070; doi:10.1186/s12870-016-0776-6)
Supplement: Additional file 7: — Description of all scenarios used in the Approximate Bayesian Computation analysis in DIYABC v2.0.4 to test the hybrid origin. (PDF 164 kb) [file 12870_2016_776_MOESM7_ESM.pdf]

Table S13 Description of all scenarios used in the Approximate Bayesian Computation analysis in DIYABC v2.0.4 to test the hybrid origin.

	Scenario	Posterior probability	Credibility interval
SSR	1	0.978	(0.9594, 0.9966)
	2	0.004	(0.0000, 0.0010)
	3	0.0216	(0.0030, 0.0420)
Nuclear genes	1	0.382	(0.0000, 0.8079)
	2	0.28	(0.0000, 0.6736)
	3	0.338	(0.0000, 0.7526)
