# Supplementary material for: Genetic origin and composition of a natural hybrid poplar Populus × jrtyschensis from two distantly related species
Source: BMC Plant Biol. 2016 Apr 18;16:89. doi: 10.1186/s12870-016-0776-6 (PMC4836070; doi:10.1186/s12870-016-0776-6)
Supplement: Additional file 9: — Summary results of one-way ANOVAs to determine the significant differences in soil nitrogen concentration at a depth of 20–40 cm for three taxa. (PDF 98 kb) [file 12870_2016_776_MOESM9_ESM.pdf]

Additional file 5 Summary results of one-way ANOVAs to determine the significant differences on soil nitrogen concentration in 20-40 cm for three taxa. (superscript a, $P$<0.05; b, $P$<0.01; c, $P$<0.001; PN, *P. nigra*; PJ, *P. × jrtyschensis*; PL, *P. laurifolia*)

| Group | PJ 1 | PJ 2 | PJ 3 | PN 1 | PN 2 | PN 3 | PL 1 | PL 2 | PL 3 |
|---|---|---|---|---|---|---|---|---|---|
| PJ 1 | 1 | $0.024^{a}$ | 0.918 | $0.000^{c}$ | $0.000^{c}$ | 0.065 | $0.000^{c}$ | $0.043^{a}$ | $0.000^{c}$ |
| PJ 2 | | 1 | $0.033^{a}$ | $0.000^{c}$ | $0.023^{a}$ | 0.617 | $0.000^{c}$ | 0.772 | $0.000^{c}$ |
| PJ 3 | | | 1 | $0.000^{c}$ | $0.000^{c}$ | 0.079 | $0.000^{c}$ | 0.055 | $0.000^{c}$ |
| PN 1 | | | | 1 | $0.046^{a}$ | $0.000^{c}$ | 0.855 | $0.000^{c}$ | $0.000^{c}$ |
| PN 2 | | | | | 1 | $0.008^{b}$ | $0.031^{a}$ | $0.013^{b}$ | $0.000^{c}$ |
| PN 3 | | | | | | 1 | $0.000^{c}$ | 0.832 | $0.000^{c}$ |
| PL 1 | | | | | | | 1 | $0.000^{c}$ | $0.000^{c}$ |
| PL 2 | | | | | | | | 1 | $0.000^{c}$ |
| PL 3 | | | | | | | | | 1 |
