# Supplementary material for: Genetic origin and composition of a natural hybrid poplar Populus × jrtyschensis from two distantly related species
Source: BMC Plant Biol. 2016 Apr 18;16:89. doi: 10.1186/s12870-016-0776-6 (PMC4836070; doi:10.1186/s12870-016-0776-6)
Supplement: Additional file 10: — Summary results of one-way ANOVAs to determine the significant differences in soil nitrogen concentration at a depth of 40–70 cm for three taxa. (PDF 173 kb) [file 12870_2016_776_MOESM10_ESM.pdf]

Additional file 6 Summary results of one-way ANOVAs to determine the significant differences on soil nitrogen concentration in 40-70 cm for three taxa. (superscript a, *P*<0.05; b, *P*<0.01; c, *P*<0.001; PN, *P. nigra*; PJ, *P.* × *jrtyschensis*; PL, *P. laurifolia*)

Group	PJ 1	PJ 2	PJ 3	PN 1	PN 2	PN 3	PL 1	PL 2	PL 3
PJ 1	1	0.054	0.000^{c}	0.000^{c}	0.796	0.000^{c}	0.000^{c}	0.000^{c}	0.000^{c}
PJ 2		1	0.048^{a}	0.000^{c}	0.130	0.000^{c}	0.000^{c}	0.000^{c}	0.000^{c}
PJ 3			1	0.000^{c}	0.003^{b}	0.000^{c}	0.000^{c}	0.000^{c}	0.000^{c}
PN 1				1	0.000^{c}	0.000^{c}	0.000^{c}	0.130	0.000^{c}
PN 2					1	0.000^{c}	0.000^{c}	0.000^{c}	0.000^{c}
PN 3						1	0.007^{c}	0.000^{c}	0.000^{c}
PL 1							1	0.000^{c}	0.000^{c}
PL 2								1	0.000^{c}
PL 3									1
