# Supplementary material for: Genetic origin and composition of a natural hybrid poplar Populus × jrtyschensis from two distantly related species
Source: BMC Plant Biol. 2016 Apr 18;16:89. doi: 10.1186/s12870-016-0776-6 (PMC4836070; doi:10.1186/s12870-016-0776-6)
Supplement: Additional file 12: — The genes and primers used in this study. (PDF 162 kb) [file 12870_2016_776_MOESM12_ESM.pdf]

Additional file 8 The genes and primers used in this study

Gene	Forward Primer	Reverse Primer	Length (bp)
Dehy	TACTGCCATGAGCGAAGATG	GGTGTGTACCTCAGCGGTCT	874
Phyto A	CCACCCAGGTCTGACAAAGT	GAGGGATATCAGTGGCTGGA	600
Phyto B	ATATGGCGAATATGGGGTCA	GGCATCCATTTCTGCATTCT	842
PAL	TGGATTGCCATCAAATCTCA	CTCTTGCGCTCTCAACCTCT	781
AREB1	GAGCTTCACAAATGACCCGTC	ACTCCCATCCCTCCACCCT	480
ERD7	ACCTCCTCCTCTCCATCCTCA	CCGCAGCAATCAACTTACCAG	872
EIN3	GGATTACAGCGATGAAGAGAT	ACTCTCCTTGGCAGTCATCTT	583
LTCOR11	GCCAAGGTTTAAGGTTTCAATT	ACAGCAAGTCCCACATGCTCT	816
