# Supplementary material for: Genetic origin and composition of a natural hybrid poplar Populus × jrtyschensis from two distantly related species
Source: BMC Plant Biol. 2016 Apr 18;16:89. doi: 10.1186/s12870-016-0776-6 (PMC4836070; doi:10.1186/s12870-016-0776-6)
Supplement: Additional file 13: — SSR Primers used in this study. (PDF 99 kb) [file 12870_2016_776_MOESM13_ESM.pdf]

Additional file 10 SSR Primers used in this study

Primer name	Forward Primer	Reverse Primer	Length	Repeats
PeuSSR_37942	TGTTGCTTTTGAATGAGAACAGC	CCTGCAATGTGCAAACAG	315	T
PeuSSR_69373	AAATCATGGAGAATAATGGCAGTC	TTCAATGCAAAAGCAGACCAC	340	A
PeuSSR_82554	CTCCTTAGTTAGGTCCCACAAA	CCATTAACGGCTTCAAGG	193	AT
PeuSSR_98348	CCTCTTCTTCACAGAATCAGAC	GGGATGAGTGAATGGGGTTTC	256	GA
PeuSSR_135688	GAGATCATGCTGTGGAAGGTAAC	ATATATTCACGGGTTGGTGAT	204	CA
PeuSSR_135862	TGTCTTGGCTTAAACCTCC	CCACTCCATTTTCCCTATCCTCTA	272	AGA
PeuSSR_149476	CTGTTACCTGGCATTCTGTATCA	TACACTGGGAGCATTAGGCAG	313	TC
PeuSSR_185039	CCACGGCAGAATCTTCCTA	GGGTACTGTTTGTTATTTTTGG	205	TA
PeuSSR_1063	AGTTAATTGCGCATGTTCTT	AAACAAACTCCAGCAAACAT	165	CA
PeuSSR_1065	TGCAATCATATATTCCTCCC	ATAAAATTACTGCGTGCCAT	156	AC
PeuSSR_114	TTAGCCATTGGATTTCATTT	CATTGCACTCTCACACATTC	112	TTC
PeuSSR_1158	ATGCACTTCCTTCCAAATTA	ATCAGTTCCTTCAGCTTCAA	225	CTG
PeuSSR_124	TTTGAGCACTTCAACTACCA	TGTCTTCCCTTAGTCACCAC	198	CAC
PeuSSR_1255	GAACCTTAAAACCAGAACCC	GAGCCACAGAAATACTGCTC	207	AG
PeuSSR_1260	CACAGGAACCTGGTTATCAT	CTGGCATTCCTTCTAAGCTA	134	TG
PeuSSR_11	CGATTAATCTCTTCTACTAGGCCATT	GCTGCTGCTAATGGATGTGA	185	AT
PeuSSR_186	GGCTAGGAATACCCTGGAGAA	AAGCCATCTCGACTATACACCA	234	TTTA
PeuSSR_190	CCCTGGTTTTCTCTTCTTGG	CCAGATTGGACTTGGGATTC	209	TG
PeuSSR_264	AAACCATAGGCATCAAAGCA	TTCATGCAAGGCATCAATTC	197	GT
PeuSSR_279	TCAAATCAAACCACAAAAACACA	TGAGACGAACATATCCTTCACC	197	AT
