# Supplementary material for: Genetic origin and composition of a natural hybrid poplar Populus × jrtyschensis from two distantly related species
Source: BMC Plant Biol. 2016 Apr 18;16:89. doi: 10.1186/s12870-016-0776-6 (PMC4836070; doi:10.1186/s12870-016-0776-6)
Supplement: Additional file 14: — The three different sites for each taxon used for the soil nitrogen analysis. (PDF 152 kb) [file 12870_2016_776_MOESM14_ESM.pdf]

Additional file 11 Three different sites for each taxon used in soil nitrogen analysis.

Taxon	popID	Latitude (◦N)	Longitude (◦W)	Altitude (M)
Populus nigra L.	Pn29	47.36342	87.82258	562
	Pn31	46.70075	87.71975	562
	Pn70	47.99892	85.69708	424
Populus × *jrtyschensis* Ch. Y. Yang	Pj36	47.26303	88.29742	578
	Pj52	47.72192	86.82469	471
	Pj60	48.14525	86.39414	565
Populus laurifolia Ledeb.	Pl12	46.85089	90.29322	1386
	Pl20	47.2465	89.9395	1274
	Pl41	47.54531	87.896	527
