# Supplementary material for: Genetic origin and composition of a natural hybrid poplar Populus × jrtyschensis from two distantly related species
Source: BMC Plant Biol. 2016 Apr 18;16:89. doi: 10.1186/s12870-016-0776-6 (PMC4836070; doi:10.1186/s12870-016-0776-6)
Supplement: Additional file 15: — GenBank Accession numbers for all newly obtained sequences for the three taxa included in this study. (PDF 17 kb) [file 12870_2016_776_MOESM15_ESM.pdf]

Additional file 9 GenBank Accession numbers of all newly obtained sequences for the three taxa included in this study.

Species	*rbc*L sequence and Nuclear genes	Genebank Accession numbers
Populus nigra L.	*rbc*L	KT626975 - KT627120
	Dehy	KT628410 - KT628497
	Phyto A	KT629436 - KT629521
	Phyto B	KT629522 - KT629603
	PAL	KT629364 - KT629435
	AREB1	KT628322 - KT628409
	ERD7	KT628566 - KT628659
	EIN3	KT628498 - KT628565
	LTCOR11	KT628660 - KT628729
Populus × *jrtyschensis* Ch. Y. Yang	*rbc*L	KT628107 - KT628321
	Dehy	KT627237 - KT627354
	Phyto A	KT627849 - KT627978
	Phyto B	KT627979 - KT628106
	PAL	KT627723 - KT627848
	AREB1	KT627121 - KT627236
	ERD7	KT627465 - KT627594
	EIN3	KT627355 - KT627464
	LTCOR11	KT627595 - KT627722
Populus laurifolia Ledeb.	*rbc*L	KT629604 - KT629820
	Dehy	KT628810 - KT628883
	Phyto A	KT629212 - KT629285
	Phyto B	KT629286 - KT629363
	PAL	KT629132 - KT629211
	AREB1	KT628730 - KT628809
	ERD7	KT628968 - KT629049
	EIN3	KT628884 - KT628967
	LTCOR11	KT629050 - KT629131
